# Mapping Evapotranspiration of Agricultural Areas in Ghana

**DOI:** 10.1155/2021/8878631

**Published:** 2021-04-17

**Authors:** Kenneth Aidoo, Nana Ama Browne Klutse, Kofi Asare, Comfort Gyasiwaa Botchway, Samuel Fosuhene

**Affiliations:** ^1^Remote Sensing and Climate Center, Ghana Space Science and Technology Institute, Accra, Ghana; ^2^Department of Physics, University of Ghana, Accra, Ghana; ^3^African Institute of Mathematical Sciences, Kigali, Rwanda

## Abstract

Climate change is having an adverse effect on the environment especially in sub-Sahara Africa, where capacity for natural resource management such as water is very low. The scope of the effect on land use types have to be estimated to inform proper remedy. A combined estimation of transpiration and evaporation from plants and soil is critical to determine annual water requirement for different land use. Evapotranspiration (ET) is a major component in the world hydrological cycle, and understanding its spatial dimensions is critical in evaluating the effects it has on regional land use. A measure of this component is challenging due to variation in rainfall and environmental changes. The mapping evapotranspiration with high resolution and internalized calibration (METRIC) method is employed to create evapotranspiration map for land use, using remotely sensed data by satellite, processed, and analyzed in ArcGIS. Normalized difference vegetation index (NDVI) was related to the availability of water for vegetation on different land use, and the results indicate a high evapotranspiration for vegetated land use with high NDVI than land use with low NDVI.

## 1. Introduction

Accurate estimate of evapotranspiration (ET) in agricultural communities in sub-Sahara Africa is important for the prudent use of water resources for agricultural purposes and the ever growing rural population [1]. ET being the sum of water movement from both plants and the soil to the atmosphere is a critical factor in agricultural decision, if agricultural activities are to have a significant impact on the economies of rural communities [2, 3]. But the ET as an important component in the water cycle has numerous challenges in its estimation, especially in the transitional zone of Ghana where there is little or no estimation for the agrarian communities to use in their water resources planning and management.

There are few instrument stations, and for those that are mounted, only a few are functioning. Coupled with the obsolete nature of the instruments makes ET estimation dare, and for that matter, the accuracy of the estimation is to be doubtful. This, however, makes the transitional zone to be the area with little or no ET research carried out. There is the need for such information for effective agricultural practices in the communities.

The functioning stations instrument can estimate ET at a field scale and thus does not take into account the spatiotemporal distribution at the regional scale, which covers the surrounding farming communities [4, 5]. To overcome this challenge, remote sensing means of estimating ET has become the viable option, and over the past decades, several methods have been developed to estimate ET at local, regional, and global scale [6, 7]. Most of these models apply reflectance from remotely sensed imagery to compute ET as a residual of energy balance at the earth's surface [8–10].

Estimating ET with the surface energy balance algorithm for land (SEBAL) method utilizes latent heat flux from remotely sensed data to make a direct assessment of actual ET. The approach of using remotely sensed data stresses the need without prior information on the crop and soil within the area of concern [11]. ET is thus determined from remotely sensed imagery by estimating the energy consumed by the ET process as a residual of surface energy.

On the basis of SEBAL, Allen et al. [8, 12] employed the mapping evapotranspiration at high resolution with internalized calibration (METRIC) model which calibrates internally with ground-based reference ET to reduce the inherent biases in the estimation of remotely sensed based ET in unison with the traditional ET methods. The goal of this study is to employ the METRIC model in estimating ET in selected farming communities where there are functioning ground base reference ET stations to aid in the spatiotemporal estimation of ET distribution.

## 2. Methods

### 2.1. Study Area

**Figure 1:**
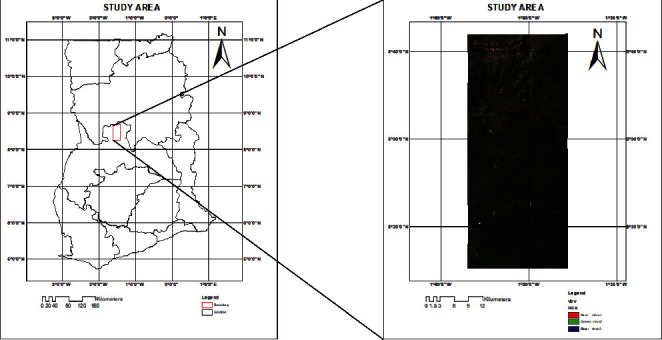
Study area.

The study area is a predominately farming community and lies along Kintampo–Buipe road and stretches from 8° 15' 17”N, 1° 36' 58”W at the lower left corner to 8^0^ 41' 56”N, 1° 25' 38”W at the upper right corner, respectively, as shown in Figure 1. It is in the transitional zone and situated between the savanna zone at the northern part of Ghana and the forest zone in the south. The vegetation of the area ranges from savanna forest in the northern part to much forested part in the south. The climatic condition is relatively moderate with an average annual temperature of 28°C and rainfall of 1345 mm. The terrain is moderately flat with a fertile soil for whole range of agricultural products.

### 2.2. Dataset

The data for the study came in two parts; the Landsat 8 products provided by the United States Geological Service (USGS) EROS Center and the packages contain all the supporting files and were processed for top-of-atmosphere (TOA) reflectance by using radiometric rescaling coefficients with the product metadata file (MTL file). The satellite imagery downloaded was in 2015, with one on February 28 and the other on November 11, thus having eight month interval to capture most of the farming activities. These were carefully selected owning to the excessive cloud cover in the study area throughout the year. A recorded, daily ET from automatic weather stations in the study area from Ghana Meteorological Agency in the same year was collected and used as a reference ET for the METRIC process.

### 2.3. Image Processing

From a remotely sensed data, corrections were made for the atmosphere by computing top-of-atmospheric (TOA) reflectance for the Landsat 8 imagery. Image classification was performed, taken into account five distinctively known land use type samples from the scene to classify the entire image of the study area, and normalized difference vegetation index (NDVI) was subsequently computed from the corrected reflectance image.

Thus,

(1) $$\begin{array}{c} \text{NDVI}=\frac{P2-P1}{P2+P1}, \end{array}$$

where *P*_2_ is the corrected reflectance in the near-infrared band 5, and *P*_1_ is the corrected reflectance in the red band 4.

Mapping evapotranspiration at high resolution with internalized calibration (METRIC) is an image-processing approach for computing instantaneous evapotranspiration (ET) as a residual of the energy balance equation [8, 12].

(2) $$\begin{array}{c} \text{LE}=R_{n}-G-H, \end{array}$$

where LE is the latent heat flux (W m^−2^), *R*_*n*_ is the net radiation (W m^−2^), *G* is the soil heat flux (W m^−2^), and *H* is the sensible heat flux (W m^−2^).

Net radiation (*R*_*n*_) is the net of radiation at the surface. It is calculated by the difference of all the outgoing radiation from all the incoming radiation and expressed in the surface balance equation.

(3) $$\begin{array}{c} \;R_{n}=R_{s↓}-\alpha R_{s↓}+R_{L↓}+R_{L↑\;}-\left(1-\varepsilon_{0}\right)R_{L↓}, \end{array}$$

where *R*_*s*↓_ is the incoming short-wave radiation (W m^−2^), *α* is the surface albedo (dimensionless), *R*_*L*↓_ is the incoming long-wave radiation (W m^−2^), *R*_*L*↑ _ is the outgoing long-wave radiation (W m^−2^), *ε*_0_ is the surface thermal emissivity (dimensionless), and (1 − *ε*0)*R*_*L*↓_ is the component of the fraction of incoming long-wave radiation.

Soil heat flux (*G*) is the amount of heat flux that is stored or released into the soil. This was determined by the expression described by [13]

(4a) $$\begin{array}{c} \frac{G}{R_{n}}=0.05+0.18e^{-0.521\text{LAI}}\;\text{LAI}\geq 0.5, \end{array}$$

(4b) $$\begin{array}{c} \frac{G}{R_{n}}=\frac{1.80\left(T_{s}-\;273.15\right)}{R_{n}}+0.084\;\text{LAI}<0.5, \end{array}$$

where LAI is the leaf area index.

Sensible heat flux (*H*) being the rate at which heat loss by air through convection and conduction as results of temperature difference is expressed by aerodynamic function.

(5) $$\begin{array}{c} H=\rho_{\text{air}}\;C_{p}\frac{d\text{T}}{r_{\text{ah}}}, \end{array}$$

where  *ρ*_air_ is the air density (kg m^−3^), *C*_*p*_ is the specific heat of air at constant pressure (J kg^−1^K^−1^), *d*T is the temperature difference between two heights *z*_1_ (0.1 m) and *z*_2_ (2 m), and *r*_ah_ is the aerodynamic resistance to heat transfer (s m^−1^).

The METRIC approach made use of internal calibration of extreme pixels of dry soil and well-irrigated agricultural fields to estimate instantaneous ET. This approach diminishes the possibility of impact of biases in the estimation of roughness and aerodynamic stability correction. The latent heat (LE) value is then used to compute instantaneous ET for each pixel in the image.

(6) $$\begin{array}{c} {\text{ET}}_{\text{inst}}=3600x\frac{\text{LE}}{\lambda \rho \omega }, \end{array}$$

where ET_inst_  is the instantaneous ET at the satellite overpass (mm h^−1^), 3600 is the conversion factor from seconds to hours, LE is the latent heat flux (W m^−2^) from equation (2), *λ* is the latent heat of vaporization = 2.26 106 (J kg^−1^), and *ρω* is the density of water (1000 kg m^−3^).

Reference ET fraction (ET_frac_) also known as crop coefficient (*K*_*c*_) was computed using ET_inst_  for each pixel and hourly reference evapotranspiration (ET_o_) acquired from weather data locally.

(7) $$\begin{array}{c} {\text{ET}}_{\text{frac}}=\frac{{\text{ET}}_{\text{inst}}\;}{{\text{ET}}_{\text{o}}}. \end{array}$$

The daily actual evapotranspiration (ET_24_) was ultimately calculated by the following expression:

(8) $$\begin{array}{c} {\text{ET}}_{24}={\text{ET}}_{\text{frac}}\;x\;{\text{ET}}_{\text{r}}, \end{array}$$

where ET_24_ is the actual evapotranspiration for the 24-hour period (mm day^−1^), ET_frac_ is from equation (7), and ET_r_ is the daily reference evapotranspiration (mm day^−1^).

Monthly and seasonal ET is derived by interpolating ET_frac_ values between images on daily basis and multiplying with ET_r_ for each day and integrates over a specific month. The ET_frac_ values from interpolation are derived by the use of spline functions, and a good cloud-free satellite image per month is ideal to determine seasonal ET_frac_ for an entire seasonal ET estimate [12, 14]. The accumulated ET for any given time is calculated as

(9) $$\begin{array}{c} {\text{ET}}_{\text{t}}=\overset{n}{\underset{i=m}{\sum }}\left({\text{ET}}_{\text{frac}i}\right)\left({\text{ET}}_{\text{r}i}\right), \end{array}$$

where ET_t_ (mm day^−1^) is the cumulative ET for a given time frame, starting with day *m* and ending with day *n*. ET_frac*i*_ is the interpolate value of ET_frac_ for day *I,* and ET_r*i*_ (mm day^−1^) is the 24-hour ET for day *i*.

Daily evapotranspiration from two weather stations in the study area was collated to represent the area as a reference evapotranspiration and subsequently applied to the reference ET fraction (ET_frac_) to ultimately estimate the evapotranspiration for the entire area. These reference ET values were computed using weather datasets and applying Penman–Monteith (PM) equation.

(10) $$\begin{array}{c} {\text{ET}}_{\text{o}}=\frac{0.408\Delta \left(R_{n}-G\right)+\gamma \left(900/T+273\right)U_{2}\left(\text{es}-\text{ea}\right)}{\Delta +\gamma \left(1+0.34U2\right)}, \end{array}$$

where ET_o_ is the reference evapotranspiration (mm day^−1^), *R*_*n*_ is the net radiation at the crop surface (MJ m^−2^ day^−1^), *G* is the soil heat flux density (MJ m^−2^ day^−1^), *T* is the mean daily air temperature at 2 m height (°C), *U*_2_ is the wind speed at 2 m height (m s^−1^), es is the saturation vapour pressure (kPa), ea is the actual vapour pressure (kPa), es − ea is the saturation vapour pressure deficit (kPa), Δ slope is the vapour pressure curve (kPa °C^−1^), and *γ* is the psychrometric constant (kPa °C^−1^).

## 3. Results

**Figure 2:**
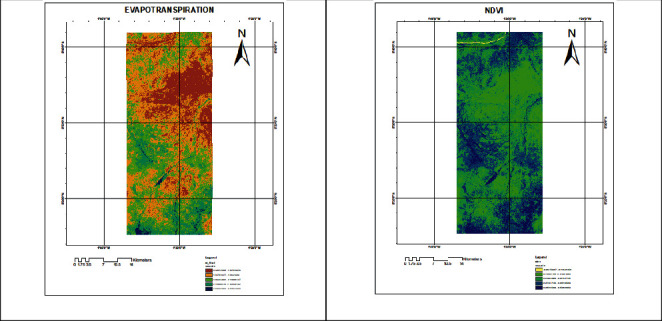
Evapotranspiration and normalized difference vegetation index map for 28/02/2015.

Spatial and temporal distribution of evapotranspiration in five land use classes with their corresponding normalized difference vegetation index for each of the satellite imagery was analyzed. It indicates that evapotranspiration is directly proportional to the normalized difference vegetation index. The results presented in Figure 2 indicate varying values for both evapotranspiration and NDVI for different land use classes, and these values were found to be relatively low in February, with ranges 0.9982–1.8819 and 0.1183–0.2519, respectively, as given in Table 1.

**Figure 3:**
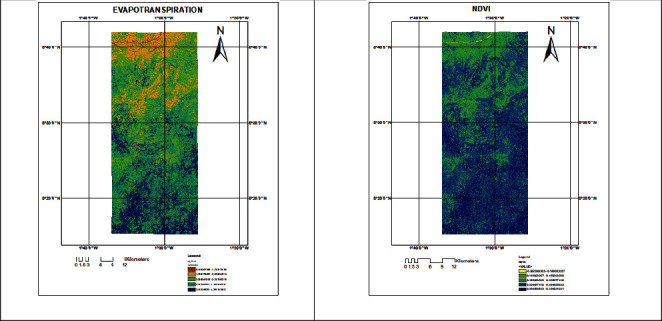
Evapotranspiration and normalized difference vegetation index map for 11/11/2015.

**Figure 4:**
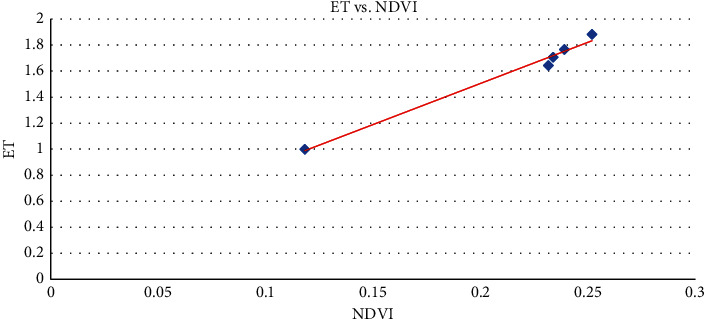
Scatter plot of ET against NDVI in February.

The results presented in Figure 3 are found to have equally varying values for evapotranspiration and NDVI for the selected land use classes, and these values were higher in November as given in Table 2 with ranges of 1.2229–2.9180 and 0.1539–0.5660, respectively. When evapotranspiration was plotted against normalized difference vegetation index (NDVI) for land use in Figure 4, a strong correlation of the ET and the normalized difference vegetation index (Pearson correlation = 0.9929768, *t* = 14.537, *d*f = 3, *p* value = 0.0007058) was found.

**Figure 5:**
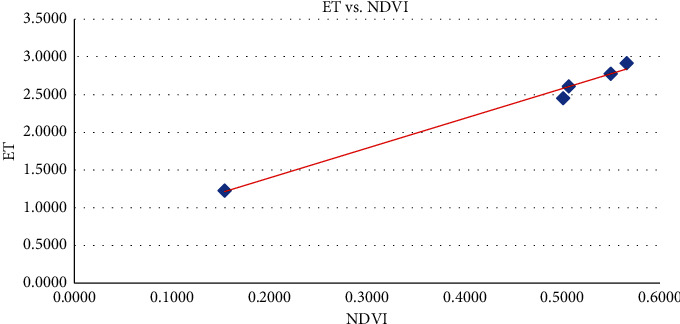
Scatter plot of ET against NDVI in November.

Also, for evapotranspiration plot against normalized difference vegetation index (NDVI) for land use classes presented in Figure 5, the trend continues with ET showing a strong correlation with NDVI (Pearson correlation = 0.9959783, *t* = 19.254, *d*f = 3, *p* value = 0.000306).

**Figure 6:**
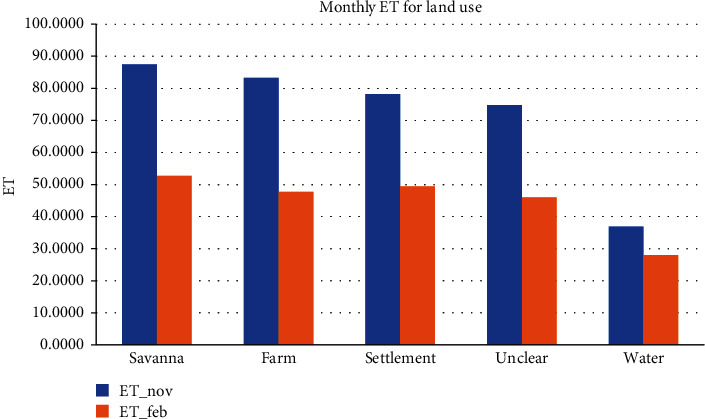
Monthly evapotranspiration.

The plot indicating monthly evapotranspiration comparison in Figure 6 indicates average monthly evapotranspiration for November in respective land use classes and also given in Table 3 are higher than that of February.

## 4. Discussion

This study used METRIC technique to investigate the pattern of evapotranspiration in different land use and the corresponding effects other indicator contributes. As was indicated, spatiotemporal distribution of evapotranspiration increases strongly with normalized difference vegetation index (NDVI) in different land uses. Thus, land use with most vegetation cover shown in Figure 2 seems to have higher values for both evapotranspiration and normalized difference vegetation index. These findings are consistent with studies [15–18] which suggest high correlation of evapotranspiration and normalized difference vegetation index (NDVI).

The results also indicated higher values for both NDVI and evapotranspiration for daily and monthly comparison for November. This was due to the fact that February happens to be in the dry season, and as a result, the soil moisture content is relatively low. Although, a comprehension of the effect of reducing soil moisture on the vegetation cover, and for that matter, NDVI is of importance. As indicated in an earlier discussion, NDVI correlates well with ET, which happens to be proportional to water vaporized into the atmosphere [17–19]. The higher values for ET and NDVI in November was due to the fact that rains usually stop at the end of October to early November, and as such, the soil moisture was relatively high and for that matter high values.

The results present evapotranspiration in respective land use class which is fundamental in getting accurate ET for water planning for agricultural purposes in respective farming communities and also for identifying particular land use for conversion. It aids in getting accurate water requirement for crops in different parts of the study area as against generalizing the entire area to have a specific ET. This finding is consistent with other studies where ET from specific fields were extracted from satellite imagery [15, 20, 21]. The result also indicates the growth stage of vegetation in land use type like farm, where high ET in November corresponds to high crop water demand for vegetable crops in their mid-season. The ET maps presented therefore highlight the variability in the crop water demand during the growing season.

The significant difference in values of ET for water between the month of November and February was attributed to increasing water plants growing in the water bodies in November thus visible in the imagery.

## 5. Conclusion

The objective was to produce ET applying the METRIC model at a high resolution considering the sparsely ground weather stations to monitor ET on a large scale. In this research, remote sensing-based ET is produced to extract ET in respective land use in the study area. It is the first of its kind to provide ET data for land use in the location with such spatiotemporal resolution. Thus, the estimated ET is expected to contribute to understanding of crop water requirement in respective farming communities for sustainable water management.

The strong correlation between estimated ET and normalized difference vegetation index for different land use takes into account the spatiotemporal distribution of the effect that makes the METRIC model ideal for large scale monitoring.

## Figures and Tables

**Table 1:** ET and NDVI values in February.

Classname	ET	NDVI
Savanna	1.8819	0.2519
Farm	1.7050	0.2338
Settlement	1.7665	0.2390
Unclear	1.6426	0.2316
Water	0.9982	0.1183

**Table 2:** ET and NDVI values in November.

Classname	ET	NDVI
Savanna	2.9180	0.5660
Farm	2.7763	0.5498
Settlement	2.6090	0.5067
Unclear	2.4920	0.5010
Water	1.2295	0.1539

**Table 3:** Monthly evapotranspiration values.

Classname	ET_Nov	ET_FEB
Savanna	87.5395	52.6925
Farm	83.2897	47.7395
Settlement	78.2715	49.4619
Unclear	74.7590	45.9916
Water	36.8841	27.9483

## Data Availability

The weather data used to support the findings of this study may be released upon request to the Ghana Meteorological Agency, and the Landsat 8 Imagery was downloaded from the website of the United States Geological Service (USGS) EROS Center.
